# The influence of responsible leadership on teachers’ green behavior: The mediating role of psychological capital

**DOI:** 10.3389/fpsyg.2023.1117386

**Published:** 2023-01-25

**Authors:** Xinyi Wang, Fengtian Kou, Kexuan Zhu

**Affiliations:** Dhurakij Pundit University, Bangkok, Thailand

**Keywords:** responsible leadership, teachers’ green behavior, psychological capital, social learning theory, Chinese university faculty

## Abstract

This research aimed to explore the impact of responsible leadership on teachers’ green behavior in Chinese university, and applied psychological capital as a mediator variable to establish a research model. A questionnaire was conducted with 303 teachers using convenience sampling. SPSS version 19 was used to analyze the data and Sobel was used to test the mediating relationships. The results show that responsible leadership has a positive yet significant effect on teachers’ green behavior. It also shows positive impact on psychological capital. Furthermore, psychological capital is shown to positively impact teachers’ green behavior, while having a mediating effect between responsible leadership and teachers’ green behavior. This study enriches the research of teachers’ green behavior and fill the gap in previous education management research. The research conclusions enable managers to better understand teachers’ green behavior and provides them with theoretical guidance for promoting psychological capital and improving teachers’ green behavior.

## 1. Introduction

The rapid development of industrial civilization has brought about the rapid growth of productivity, but it has also brought about many environmental problems: ecosystem degradation is severe, biodiversity is sharply reduced, and natural disasters occur frequently. These phenomena have aroused people’s wide concern for environmental problems (Inauen et al., 2021) and also affect human health and well-being (Evans, 2019). Over the past few decades, organizations have been proactively and comprehensively addressing environmental issues (Wolff et al., 2018), as there is growing concern about the long-term antagonistic effects of climate change and environmental degradation (Aguinis and Glavas, 2012). Past research has shown that green behavior among employees can help improve the environment (Zhen et al., 2002; Unsworth et al., 2021). Employee green behavior is any personally assessable behavior that can contribute to environmental sustainability in the workplace (Andersson et al., 2013). Therefore, employee green behavior has become a kind of behavior that organizations and employees work together to help the organization and the environment (Chaudhary, 2020; Tian et al., 2020). The vision and mission of for-profit and non-profit organizations differ greatly in terms of underlying motivations, one being primarily revenue driven and the other being social mission-driven (Quarter and Richmond, 2001). Thus, the mechanisms that influence employee green behavior in education may be very different from those in business but have never been explored.

Employee green behavior is a kind of positive organizational behavior, which is considered a micro activity to solve the problems of the environment and sustainable development, and is pro-social (Zhang et al., 2021). This can be demonstrated through recycling, rational use of resources, participation in environmental activities, and the maintenance of sustainable policies (De Roeck and Farooq, 2018). Based on social learning theory (Bandura, 1986), leadership is regarded as an important antecedent variable affecting employees’ green behaviors, because leaders, as representatives of an organization, exert a profound influence on employees through their words and deeds (Afsar et al., 2020). Several studies have shown that leadership style can influence employees’ green behavior, such as ethical leadership (Ahmad et al., 2021), servant leadership (Ying et al., 2020), and taoist leadership (Xing and Starik, 2017). However, the above studies are based on the binary relationship between leaders and employees, which fails to respond positively to the needs of stakeholders and is not fully consistent with the social responsibility and ethical values of the organization (Tian and Suo, 2021).

Responsible leadership is a powerful complement to existing research frameworks on leadership traits and leadership theories and can address scandals at the individual, organizational and system levels, as well as ethical and environmental challenges arising from new social and environmental issues (Pless and Maak, 2011). It is defined as the type of leadership that maintains mutual trust and collaboration between internal and external stakeholders of an organization in order to mobilize the cooperation of different stakeholders and achieve a common vision for the business (Maak and Pless, 2006). From a stakeholder perspective, responsible leadership is a hybrid of social responsibility, ethics, and leadership (Antunes and Franco, 2016; Waldman et al., 2019). Responsible leaders enhance employees’ awareness of the organization’s social responsibility and encourage them to participate in the organization’s social responsibility activities (Voegtlin et al., 2012). Also, by participating in green behavior, employees are responding to the organization’s call for social responsibility. Therefore, one of the motivations for this study was to explore the effects of responsible leadership on teachers’ green behaviors.

However, not all employees can learn and imitate the behavior of their superiors, and previous studies have not focused on the motivation resources at the individual level. The role model effect of leaders may also be affected by individual psychological factors (Bouckenooghe et al., 2015). Psychological capital has become an important part of the research on positive organizational behavior (Luthans et al., 2010). It represents a major personal motivational tendency that accumulates through positive mental constructs such as efficacy, optimism, hope, and resilience (Luthans et al., 2007). Responsible leadership requires employees to respond to the social responsibility of the organization by giving clear and transparent expected goals (Voegtlin et al., 2012), which increases followers’ motivation for positive behavior in the form of increased efficiency, hope, optimism, and resilience (Luthans et al., 2007). Therefore, the use of psychological capital as a potentially important mediating variable is of great importance for the research exploring the relationship between responsible leadership and teachers’ green behavior. As such, the second motivation for this study was to explore the mediating role of psychological capital between responsible leadership and teachers’ green behavior.

Based on the above discussion, the main contribution of this study is to explore the relationship between responsible leadership and teachers’ green behavior based on social learning theory, and verify the mediating role of psychological capital, to fill the gap in previous education management research.

## 2. Theories and hypotheses

### 2.1. Social learning theory

Social learning theory assumes that most human behavior is observed through modeling (Decker, 1986). Individuals can learn appropriate behavior and norms by observing the behavior of others, especially those that seem reasonable (Bandura and Walters, 1977). Also, according to social learning theory, the extent to which individuals see others as role models and imitate them depends on the power and status of others (Manz and Sims, 1981). Responsible leadership focuses on the interests of various stakeholders related to the organization’s business and exchanges information and opinions when communicating with employees (Witt and Stahl, 2016). In this interactive process, the leader conveys his views and insights to the employees, and the employees gradually accept and internalize the leader’s values by observing and imitating the words and deeds of the leader (Han et al., 2019). Leaders are the key objects for employees to observe in an organization (Tian and Suo, 2021). According to the research by Voegtlin et al. (2012), responsible leaders set a positive example for employees by focusing on all stakeholders. Responsible leadership provides ethical role models for employees by emphasizing the ethics of the leader and the behavior that follows ethical principles (Shi and Ye, 2016). Thus, responsible leaders can reduce unethical behavior among employees (Voegtlin, 2011) and conversely increase ethical behavior. In education, responsible headmasters should build trusting and ethical relationships with their stakeholders, for the success of the school and for the common good of the local community (Oplatka, 2017). As McCullough (2012) maintained, responsible headmasters need to build and maintain an organizational culture that is based on and fully supported by a full network of middle managers, teachers, parents, students and other stakeholders. In addition, responsible leaders care about their subordinates and when teachers feel the attention of leaders, positive psychological capital will be triggered, and they may take the goals of the organization as their own and strive to achieve them (Tian and Suo, 2021). Therefore, teachers will learn from responsible leaders and actively put into behavior in order to meet the goals and requirements of the school.

### 2.2. Responsible leadership and teachers’ green behavior

Responsible leadership is defined as “a relational and ethical phenomenon that occurs in the social process of interaction with those affected by leadership and is closely related to the purpose and vision of leadership” (Maak and Pless, 2006). As an intrinsically normative approach to leadership, responsible leadership differs from other value-centered leadership theories, such as ethical leadership (Shakeel et al., 2019), service-oriented leadership (Eva et al., 2019), authentic leadership (Whitehead, 2009), and transformational leadership (Korejan and Shahbazi, 2016). The key difference between them and responsible leadership is that responsible leadership focuses primarily on social and environmental goals, as well as goals for sustainable value creation and positive change. Responsible leaders, like weavers, have the advantage of bringing stakeholders together (Maak and Pless, 2006). Responsible leaders care about the interests of domestic and foreign stakeholders, fulfill corporate social responsibilities, and encourage employees to participate in corporate social responsibility activities (Voegtlin et al., 2012). Responsible leaders believe they have an obligation to serve and be accountable to their stakeholders, including the well-being of future generations, and continually seek the desired goal of meeting the needs of a broad range of stakeholders by focusing on virtuous outcomes (Oplatka, 2017).

Employee green behavior is one of several strategies that organizations follow to improve environmental performance and achieve sustainable development goals (DuBois and Dubois, 2012). It is defined as any evaluable behavior of an individual that contributes to environmental sustainability in the workplace (Andersson et al., 2013). Ones and Dilchert (2012) state that to achieve ecological sustainability, we need to promote, influence, and change employee behavior in a way that aligns it with the environmental sustainability goals of the organization. They refer to these environment-related employee behaviors as employee green behaviors and define them as “scalable behaviors of employee participation” (Ones and Dilchert, 2012). In addition, Stern (2000) explained employee green behavior as employees’ intentional behavior to help reduce negative human behavior. It may include water conservation, efficient use of resources, waste reduction, energy conservation, and recycling (Norton et al., 2015).

Research shows that leadership style is closely related to employees’ green behaviors (Wang et al., 2018, Ahmad et al., 2021, Hameed et al., 2022). According to social learning theory (Bandura, 1986), subordinates guide their behavior by observing, imitating, and internalizing the values of the leader, leading to the replication of the leader’s behavior. There is a positive correlation between leaders’ environmental behaviors and those of their subordinates (Robertson and Barling, 2013) because leaders’ behaviors reflect their values, and leaders pass on their values to their subordinates through role models. Leaders may communicate why sustainability is important, clarify organizational direction, and set goals (Banerjee et al., 2003; Colwell and Joshi, 2013; Young et al., 2015). Their actions will increase employees’ focus on sustainability (Banerjee et al., 2003; Colwell and Joshi, 2013). Under the guidance of responsible leaders, employees will realize and understand the importance of employees’ pro-environment behaviors by imitating, learning, and following leaders (Steg and Vlek, 2009), thus increasing employees’ green behaviors.

Based on the above consideration, this study proposes research hypothesis

*H1*: responsible leadership has a positive and significant effect on teachers’ green behavior.

### 2.3. Responsible leadership and psychological capital

One form of strategic resource that has received increasing attention in the literature due to its impact on human performance is psychological capital (Ardichvili, 2011). Like human capital, psychological capital can be viewed as an asset that organizations need to embrace, develop and manage to achieve effective work behavior and organizational outcomes (Froman, 2010). It is defined as a positive state of individual psychological development and consists of four components: self-efficacy, hope, optimism, and resilience (Luthans and Youssef, 2007). Self-efficacy is defined as a person’s belief or confidence in his or her motivation, cognitive resources, or ability to successfully perform a specific task in a given setting (Stajkovic and Luthans, 1998); Optimism refers to an individual’s expectation of a positive outcome (Scheier et al., 2001); Hope is defined as a positive motivation based on an interactivity-derived state based on two aspects: agency (goal-directed energy) and path (a plan to achieve a goal; Snyder et al., 1996); Resilience refers to the ability to bounce back or recover from adversity, conflict, failure or even positive events, progress and increased responsibility (Luthans, 2002).

As an important environmental variable in an individual’s psychological capital, responsible leadership may affect psychological capital. Research has shown that leaders are a major source of both positive and negative emotions for employees at work (Dasborough and Ashkanasy, 2002). Doh and Quigley argue that responsible leaders increase employees’ trust in leaders by demonstrating their responsible and guided actions, which may bring significant benefits to the organization and relevant stakeholders, such as employee development of positive psychology (Doh and Quigley, 2014). According to social learning theory (Bandura and Walters, 1977), the example of a leader can serve as a clear road map, constitute desirable behaviors toward the realization of goals, and help employees establish positive mental states and necessary resources to perform well at work (Gardner et al., 2005; Gooty et al., 2009). Responsible leaders are very noble, do-good oriented leaders, a type of leadership that has a sense of justice, recognition, responsibility and concern for others (Cameron, 2011). A responsible leader is a good role model for employees and can help to generate more positive psychology.

Based on the above consideration, this study proposes research hypothesis

*H2*: responsible leadership has a positive and significant effect on psychological capital.

### 2.4. Psychological capital and teachers’ green behavior

The construction of psychological capital can be used to capture individual positive behavior. Individuals with high self-efficacy are driven by confidence in their ability to successfully perform certain tasks (Miao et al., 2018) and are also motivated by their behavior or the expected outcome of their behavior (Bandura, 1999). Optimistic people tend to expect positive outcomes from their actions (Bak et al., 2022). Researchers believe that resilience is related to employee behavior toward organizational development and sustainability in this rapidly changing era of globalization, where employees not only need to cope, and need to successfully recover from uncertainty, difficulties, and major changes (Luthans et al., 2007; Quick and Feldman, 2014). Also, hopeful employees are more likely to actively pursue goals, develop different ideas, and generate alternative pathways (e.g., green action plans) to achieve them (Luthans et al., 2007; Sweetman et al., 2011; Rego et al., 2012). Therefore, all four dimensions of psychological capital contribute to the positive behavior of employees. A Bangladeshi study found that employees with higher levels of positive psychological capital were more likely to engage in environmentally responsible behavior in the workplace (Afshar Jahanshahi et al., 2021). In summary, people with positive psychological capital are more likely to go beyond their regular tasks in the workplace and engage in voluntary, context-driven behavior.

Based on the above consideration, this study proposes research hypothesis

*H3*: psychological capital has a positive and significant effect on teachers’ green behavior.

### 2.5. The mediating role of psychological capital

In organizations, people with higher levels of psychological capital show better work outcomes than those with lower levels of psychological capital (Newman et al., 2014). Employees with low psychological capital are more likely to produce negative work outcomes, such as turnover intention, etc. (Zhu et al., 2022). The reason may be that individuals with high self-efficacy adjust their goals according to their beliefs about their abilities, and therefore put more effort into achieving them (Seo and Ilies, 2009, Bandura, 2012). Also, more optimistic people receive more professional and psychosocial support throughout their careers than those who are less optimistic (Higgins et al., 2010). Responsible leaders actively participate in social responsibility activities (Shi and Ye, 2016), such as providing a comfortable working environment for teachers, establishing a good learning environment for students, educating and raising children for parents, working fairly and honestly with other units, saving resources, and protecting the environment. When teachers feel supported by their leaders, positive emotions are triggered, and they are more motivated to take environmental measures to help the school achieve sustainable development goals and create a long-term competitive advantage (Tian and Suo, 2021).

Many studies have confirmed the mediating role of psychological capital in the relationship between leadership and employee behavior. For example, a study in Sri Lanka confirmed the mediating role of psychological capital between authentic leadership and organizational citizenship behavior (Ramalu and Janadari, 2020). An Indian study showed that psychological capital had a significant mediating effect between sincere leadership and additional role behavior of nurses (Malik and Dhar, 2017). A Turkish study supported the mediating role of psychological capital in the relationship between ethical leadership and service innovation behavior (Özsungur, 2019).

Based on the above consideration, this study proposes research hypothesis

**Figure 1:**
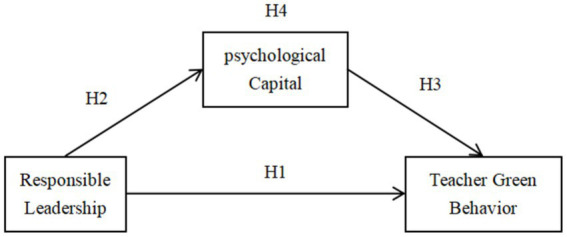
Research framework.

*H4*: psychological capital has a mediating role between responsible leadership and teachers’ green behavior (Figure 1).

## 3. Research methods

### 3.1. Participants and procedures

The questionnaire was conducted for 2 months from September 2022 to November 2022. The study collected feedback from Chinese university faculty. HR heads of the schools were approached *via* emails and phones for the purpose of data collection. After a discussion on the purpose, procedure, anonymity and confidentiality of the study, some of the HR heads agreed to the request and asked the author to mail them the link to online questionnaire, which they circulated among their teachers. Questionnaires are distributed on a convenience basis. The main reason to use convenience sampling is the hectic schedule of such respondents.

Referring to Tinsley and Tinsley (1987), the number of questionnaires issued should be combined with the number of questions; the ratio of the number of items to the sample size should be between 1: 5 or 1:10. There are 28 items in this survey, and the maximum ratio is 1:10. So at least 280 valid samples are needed for this study. On the other hand, multiple regressions with sample sizes of 200–500 are valid, which may be used for more rigorous impact assessments (Ahmed et al., 2011).

In consideration of the possibility that some questionnaires might not be valid, a total of 320 questionnaires were distributed. Based on the screening of negative questions, invalid questionnaires were eliminated and 303 valid questionnaires were finally collected. To ensure the validity of the questionnaire, invalid questionnaires such as incomplete information were eliminated. Finally, 303 valid questionnaires were collected. The proportion of valid questionnaires was 94.69%. Among them, 123 cases were male, accounting for 40.60%; 180 cases (59.40%) were female. In terms of age, 91 people were between 20 and 29 years old, accounting for 30.00%; 139 people aged 30–39, accounting for 45.90%; 57 people aged 40–49, accounting for 18.80%; 16 people aged 50 and above, accounting for 5.30%.

In order to evaluate the common method variance in this study, we ran the Harman’s single-factor test. The results showed that 7 factors can explain the majority of variance (the maximum component explained only 31.681% of total variance), which means that there was no common method bias in this study.

### 3.2. Measures

In this study, the mature scale widely used was used to measure variables, and the questionnaire items were scored by the Likert5 score system. 1 means “strongly disagree” and 5 means “strongly agree.” The higher the number, the higher the level of recognition.

- Responsible Leadership: the scale developed by Voegtlin (2011) consists of five items. Measures include “My superiors indicate that they are aware of stakeholder interests” and “My superiors fully consider the outcome of stakeholder decisions.” In the study with Chinese subjects, Cronbach’s alpha coefficient was 0.847 (Han et al., 2019). In this study, the consistency reliability coefficient of the scale is 0.707.
- Teachers’ green behavior: using the Workplace environmentally-friendly Behavior Scale developed by Robertson and Barling (2013), which is a one-dimensional structure with seven items. For example, “I print double-sided whenever possible,” “I turn off the lights when not in use,” etc. The consistency reliability coefficient of this scale is 0.757.
- Psychological capital: using the scale developed by Luthans et al. (2006), the scale is a four-dimensional structure with 16 items. Sample items in the scale included: “I now consider myself fairly successful at work,” “I can think of many ways to get out of difficult situations at work,” and “I always look on the bright side of things at work.” In this study, the consistency reliability coefficient of the scale was 0.926.
- Control variables: demographic variables. In this study, teachers’ gender and age were used as control variables.

### 3.3. Statistical analysis

Firstly, SPSS 22.0 software was used to conduct descriptive statistics and Pearson correlation coefficient analysis for responsible leadership, teachers’ green behavior, and psychological capital variables. Finally, we explore the specific relationship between the three variable pairs and examine the mediating role of psychological capital in the influence of responsible leadership on teachers’ green behavior. Finally, this study uses Sobel for mediation verification.

## 4. Results

### 4.1. Descriptive statistics and correlation analysis

Descriptive statistics show that college teachers’ perception of responsible leadership, teachers’ green behavior, and psychological capital are all at an above-average level. As can be seen from Table 1, there is a significant positive correlation between responsible leadership and teachers’ green behavior (*r* = 0.307, *p* < 0.001). There was a significant positive correlation between responsible leadership and psychological capital (*r* = 0.171, *p* < 0.01), and there was a significant positive correlation between psychological capital and teachers’ green behavior (*r* = 0.326, *p* < 0.001). The correlation coefficient is 0.171 ~ 0.326, without collinearity.

**Table 1:** Variable descriptive statistics and correlation analysis.

Variable	*M*	SD	RL	GB	PC
RL	3.709	0.469	1		
GB	4.022	0.579	0.307^***^	1	
PC	3.976	0.518	0.171^**^	0.326^***^	1

^***^p < 0.001, ^**^p < 0.01, ^*^p < 0.05. RL, Responsible Leadership; GB, Green Behavior; PC, Psychological Capital.

### 4.2. Regression analysis

Multiple regression analyzes serve to verify the hypothesis. By controlling the influence of gender and age, the mediating effect of psychological capital on the perception of responsible leadership and teachers’ green behavior was examined.

As shown in Table 2, college teachers’ perception of responsible leadership has a significant positive impact on teachers’ green behavior (*β* = 0.281, *t* = 5.239, *p* < 0.001) in Model 1, so hypothesis H1 is valid. In Model 2, college teachers’ perception of responsible leadership had a significant positive effect on psychological capital (*β* = 0.217, *t* = 3.906, *p* < 0.001), and thus hypothesis H2 is valid as well. In Model 3, after adding the mediating variable psychological capital, responsible leadership has a significant positive effect on teachers’ green behavior (*β* = 0.221, *t* = 4.196, *p* < 0.001), and psychological capital has a significant positive effect on teachers’ green behavior (*β* = 0.273, *t* = 5.077, *p* < 0.001) and thus the validation of hypothesis H3 can be confirmed. The *β* value of the influence of college teachers’ perception of responsible leadership on teachers’ green behavior decreased from 0.281 in model 1 to 0.221 in model 3, which was at a significant level. It can be seen that psychological capital plays a partial mediating role in the influence of responsible leadership on the green behavior of college teachers, and it can be confirmed that hypothesis H4 is valid.

**Table 2:** Mediating effect of psychological capital on the relationship between responsible leadership on teacher green behavior.

Variable	Model 1		Model 2		Model 3		VIF
	GB		PC		GB		
	*β*	*t*	*β*	*t*	*β*	*t*	
Male	0.016	0.303	0.225	4.030^***^	−0.045	−0.849	1.147
20–29	−0.118	−1.051	−0.217	−1.863	−0.059	−0.541	4.757
30–39	0.015	0.130	0.102	0.828	−0.012	−0.109	5.280
40–49	0.285	2.851^**^	0.016	0.154	0.281	2.922^**^	3.750
RL	0.281	5.239^***^	0.217	3.906^***^	0.221	4.196^***^	1.129
PC					0.273	5.077^***^	1.173
*R* ^2^	0.207		0.147		0.270		
Adj *R*^2^	0.194		0.133		0.256		
*F*	15.498^***^		10.261^***^		18.288^***^		

^***^p < 0.001, ^**^p < 0.01, ^*^p < 0.05. *β* is the standardized regression coefficient. RL, Responsible Leadership; GB, Green Behavior; PC, Psychological Capital. Gender and age are dummy variables. Males are the experimental group within the gender group, while the females are the reference group. 20–29, 30–39, and 40–49 are the experimental group in the age group, while ≥ 50 are the reference group.

In this study, the Sobel test (Sobel, 1982) was used to further test the mediating effect and calculate the non-standard regression coefficient and standard deviation. If *Z* is greater than 1.96, the mediating effect is significant. The results show that *Z* = 3.103, *p* < 0.001, indicating that psychological capital plays an intermediary role in the relationship between responsible leadership and teachers’ green behavior. In Model 3, the VIF is between 1.129 to 5.280 (which is below the standard value of 10). This result indicates a lack of serious collinearity problems.

## 5. Conclusion and discussion

### 5.1. Conclusions of the study

This study aims to explore the influence mechanism of responsible leadership on college teachers’ green behavior and empirically test the mediating role of psychological capital. The results show that responsible leadership has a positive effect on teachers’ green behavior; responsible leadership has a positive influence on psychological capital; psychological capital has a positive effect on teachers’ green behavior; psychological capital plays a partial mediating role between responsible leadership and teachers’ green behavior.

### 5.2. Theoretical contributions

First, this study explores the relationship between responsible leadership and teachers’ green behavior. Due to the increasingly prominent environmental problems and the country’s gradual emphasis on green development, the academic circle mainly focuses on the green behavior of enterprise employees, but there is a lack of relevant research on teachers. Teachers not only play the role of school employees but also shoulder the important responsibility of educating students. Teachers’ independent environmental awareness and actions not only play a vital role in the sustainable development of schools but also play a role model for students, which is worthy of further discussion. Taking teachers’ green behavior as the result variable, this study verified the positive impact of responsible leadership on teachers’ green behavior, which enriched relevant research on teachers’ green behavior.

Secondly, the internal mechanism of responsible leadership affecting teachers’ green behavior is discussed, and the mediating role of psychological capital is determined. This broadened the research scope of psychological capital and enriched the research of positive psychology. At the same time, psychological capital is an intermediary variable to explore the path of responsible leadership on teachers’ green behavior, which is helpful to unlock the black box of the relationship between these two roles.

Thirdly, this study analyzes the relationship among responsible leadership, psychological capital, and teachers’ green behavior in the Chinese context. Although China has experienced decades of modern civilization, the relationship with the leader is still the most important interpersonal relationship at work, which is considered to conform to the historical ruler-subject relationship (Wei et al., 2010). Therefore, the impact of leadership style on employee behavior becomes more important in the Chinese context.

### 5.3. Practical implications

First, responsible leadership can induce teachers’ environmental awareness and environmental behavior. Therefore, to encourage teachers to show more green behaviors in their work and improve environmental performance, schools should cultivate more responsible leaders and enhance their sense of social responsibility through regular training. In order to effectively implement green initiatives, schools should provide green training to teachers to make them aware of the importance of green management and equip them with the skills and expertise needed to successfully fulfill their green responsibilities.

Second, schools should try to choose candidates with a strong sense of responsibility and environmental awareness when recruiting. Schools can examine teacher candidates’ attitudes toward green environmental protection, their understanding of social responsibility, and their daily green behaviors.

Third, psychological capital plays an intermediary role between responsible leadership and teachers’ green behavior. Therefore, in management practice, responsible leaders should actively participate in environmental activities, instill environmental concepts in teachers, and lead by example. At the same time, leaders should invest and develop teachers’ psychological capital in specific ways according to the characteristics of investment and profitability of psychological capital to tap the potential of teachers. Improve teachers’ psychological capital, to increase teachers’ initiative and enthusiasm in participating in environmental activities.

### 5.4. Limitations and prospects

Although this study has enriched the research on responsible leadership and teachers’ green behavior, it still has some shortcomings. First, this study used cross-sectional data to confirm the causal relationship between variables, but the explanatory power is not as strong as that of longitudinal research, which can be used for further research in the future. Second, this study only examined the mediating role of psychological capital, and future studies can further explore other possible mediating mechanisms between responsible leadership and teachers’ green behavior, as well as the changes in the relationship between the two in different situations. Finally, this study pays more attention to the green behaviors of employees in enterprises. Future studies can try to explore the influencing mechanism of green behaviors of employees in different industries.

## Data availability statement

The raw data supporting the conclusions of this article will be made available by the authors, without undue reservation.

## Ethics statement

The studies involving human participants were reviewed and approved by Dhurakij Pundit University. The patients/participants provided their written informed consent to participate in this study.

## Author contributions

KZ designed the study, analyzed the data, and drafted the manuscript. XW and FK assisted in analyzing and interpreting the data. All authors contributed to the article and approved the submitted version.

## Conflict of interest

The authors declare that the research was conducted in the absence of any commercial or financial relationships that could be construed as a potential conflict of interest.

## Publisher’s note

All claims expressed in this article are solely those of the authors and do not necessarily represent those of their affiliated organizations, or those of the publisher, the editors and the reviewers. Any product that may be evaluated in this article, or claim that may be made by its manufacturer, is not guaranteed or endorsed by the publisher.
